# Young children’s screen habits and first-time parents’ reflections on screen use in socioeconomically disadvantaged Swedish settings: a mixed methods study

**DOI:** 10.1186/s12889-024-19557-9

**Published:** 2024-07-29

**Authors:** Kirsi Tiitinen Mekhail, Lisa Blom, Lise-Lott Rydström

**Affiliations:** 1 Department of Neurobiology, Care Sciences and Society (NVS), Karolinska Institutet, Stockholm, Sweden; 2 Department of Global Public Health (GPH), Karolinska Institutet, Stockholm, Sweden

**Keywords:** Screen time, Developmental health, Disadvantaged population, Early childhood, Health behaviours

## Abstract

**Background:**

Despite WHO’s recommendation of limited screen time for children < 2 years, they are worldwide established screen users. Several negative consequences are connected to excessive screen use among children. As parents are key actors in introducing screens to children, it is important to explore children’s screen habits and parents’ perspectives on screen in different populations, which could support the development of guidelines for healthier screen use. This study aimed to explore young children’s screen use habits and describe first-time parents’ reflections on children’s screen use in socioeconomically disadvantaged Swedish settings.

**Methods:**

This mixed methods study was a part of larger studies within Stockholm County. The data were collected through semi-structured questionnaire-based interviews, among first-time parents (*N* = 386) of 15–18 months-olds at local Child Health Care centres during 2019–2022. Quantitative and qualitative data analyses conducted in parallel resulted in descriptive statistics and qualitative categories.

**Results:**

Most children (92.4%) used screens by the age of 15–18 months, commonly for a maximum of 30 min (48.5%) per day. Participants stated the most suitable age for starting screen use to be after (41.7%) or at (37.9%) the age of two years. Parents’ reflections on screen use formed three main categories, each having two sub-categories: *screen use patterns* (screen-related time and reasons for screen use), *perceived concerns with screen use* (child development and social issues), and *attitudes related to screen use* (parents’ attitudes towards screens and child’s response to screens).

**Conclusions:**

First-time parents in socioeconomically disadvantaged settings expressed awareness of possible negative screen-related effects and recommendations but did not always focus on long-term consequences when using screens in everyday life. Screens used as a short distraction, time spent together with screens, infants’ contact with relatives overseas, and pedagogical children’s programs and apps can be regarded as screen-related benefits. Encouraging parents’ self-reflection on their screen use may be a way of contributing to healthier screen habits among young children. Future studies are suggested regarding parents’ and children’s excessive interest in screens and how to manage screen use as a family.

**Trial registration:**

Retrospectively registered 18 February 2020 (ISRCTN10336603) and 24 May 2021 (ISRCTN66190787) in ISRCTN registry.

**Supplementary Information:**

The online version contains supplementary material available at 10.1186/s12889-024-19557-9.

## Background

Screen use, including the use of TV, smartphones, and tablets, has become an inseparable part of our everyday lives. Several studies from various parts of the world have reported that even young children under the age of two years have established daily screen use habits [1–3], and screen use from as early as the age of four months has been reported in the literature [4]. Screen use also increased during the COVID-19 pandemic, during which primary-aged children and children up to five years old experienced the greatest increase [5].

Screen use is a hot topic for debate, as research has shown that excessive screen use by children is associated with several unwanted health conditions, including obesity/overweight and shorter sleep duration among toddlers and preschoolers [6]. Associations are also found in the same age groups between screen use and other physical, behavioural, and psychosocial aspects, including aggressive behaviours, increased risk for musculoskeletal pain and bullying in the following years, poorer healthy dietary behaviour, reduced executive function and motor development, less physical and more sedentary activities, and poorer behavioural and emotional outcomes [6]. A Canadian study revealed that screen use exceeding one hour a day among preschool children harmed five developmental health domains—physical health and wellbeing, social competence, emotional maturity, language and cognitive development, and communication skills—compared to children with less than one hour of screen use per day [7].

The guidelines from the World Health Organization (WHO) regarding screen use emphasize no screen time for infants < 1 year of age and no more than an hour for 1–2 years, with less time preferred [8]. In Sweden, there are no official recommendations regarding screen time for children [9], and according to a group of researchers, Sweden is the only country in the world that recommends screen use in the preschool curriculum [10]. However, due to increased screen use among children in Sweden, the Swedish government tasked the Public Health Agency of Sweden in 2023 to develop guidelines on young people’s media use [9]. Furthermore, the Swedish Paediatric Society has recently published recommendations regarding screen use for young children, and according to their recommendations, screens should be avoided for children under two years of age [11]. To date, the guidelines for nurses working within Swedish Child Health Care (CHC) services do not include a specific time limit for children’s screen use but address the importance of other activities for children’s development and health [12]. The guidelines also describe that screens can be used to distract children during short periods of time to facilitate daily life [12].

A systematic review and meta-analysis on how well parents of young children follow screen time recommendations reports that less than 25% of parents of under two-year-olds are following the given guidelines [1]. Previous studies reveal that, according to parents, guidelines regarding screen time recommendations by healthcare professionals are not always available [13], and the lack of consensus on recommendations parents receive is regarded as one of the obstacles to managing children’s screen time [7].

As parents play a key role in all areas of young children’s lives, they also have numerous motives and thoughts about the screen use of their young children. A systematic review and meta-analysis of qualitative studies exploring parents’ perceptions of screen time for children under 12 years reports varied reasons behind the screen time described by parents [14]. These reasons include baby-sitting, educational purposes, and the reward and punishment of children [14]. This review summarizes parents’ different attitudes towards screen time under two main themes: that parents consider screen time to be a necessity and/or that they are concerned about the impact of screen time on their children’s health and development [14]. Regarding strategies and approaches to managing children’s screen time in the abovementioned review, parents described both rules and restrictions, their striving for balance, and how they faced different obstacles and recommendations [14]. An autoethnographic study by a first-time mother illuminates her struggle, coloured by the recommendations of zero screen time for young children because of its negative effects and the reality that screens are ingrained in daily routines and ways of life and cannot be removed [15].

Screen time among preschool children is found to be associated with parents’ own screen time and access to screens in bedrooms [16]. A large study from the Netherlands among families with children 0–7 years of age revealed several associations between children’s media use and household characteristics, such as educational level, income, number of screens at home and in children’s bedrooms, marital status, and number of children at home [17]. Media use varied from 1 to 6.5 h per day [17], where the highest media use was found among households with the lowest income and educational levels and among those who had the most screens at home [17]. The group with the highest media use was concerned about media use, and their children tended to use video content, educational content and print media less cognitively skilled [17]. Regarding screen use among families with a low social position, a small study in Australia found parental self-efficacy, physical resources at home, and temporal priorities in challenges of everyday life to be crucial reasons behind screen use at mealtimes in socioeconomically disadvantaged settings [18].

As young children’s screen use is a reality of our time and parents are key actors in introducing screens to children, it is important to learn more about parents’ perspectives on children’s screen use. Furthermore, as children’s screen use is related to families’ socioeconomic backgrounds, there is a need to gain a better understanding of how screens are used and how parents think about screen use in different contexts to offer parental support and guidelines leading to healthier screen use among young children.

## Methods

### The aim

The aim of this study is to explore young children’s screen use habits and describe first-time parents’ reflections on children’s screen use in socioeconomically disadvantaged Swedish settings.

### Design

The study has a mixed methods design using quantitative and qualitative data analyses of the same data material.

### Setting of the study

The study was conducted in socioeconomically disadvantaged settings in Stockholm County, Sweden. The area is characterized by a lower socioeconomic and higher care needs index (CNI) than the county average. With respect to the higher CNI, CHC centres in seven of the 10 areas included in this study were offered an expanded postnatal home visiting programme. Home visits were conducted by CHC nurses and parent counsellors from social services to improve children’s health and well-being [19]. The extended home visit program after birth has been implemented since 2013 as a supplementary intervention within the Swedish CHC program [19]. In Sweden, CHC centres offer all families a universal CHC programme that monitors children’s health and development and implements the vaccination programme among children aged 0–5 years [20]. The data analysed in this study are from two larger studies in which the effect of the extended home visiting program in the Stockholm region during 2017–2022 was evaluated. One of the studies has generated two previous publications [21, 22] and a third one is under revision.

### The characteristics of participants

Participants in this study were first-time parents who had registered their first child at any of the CHC centres located in the selected geographical areas during the recruitment periods for the two abovementioned evaluation studies (2017–2019) and who consented to participate. All the participants gave their written informed consent to participate at the time of the recruitment when the baseline interviews of the studies were conducted (in 2017–2019).

The sociodemographic background characteristics of the participating parents were collected through the questionnaire-base baseline interviews when parents were recruited for the two above mentioned studies in Stockholm region and are displayed in Table 1. The questionnaires were developed specifically for the purpose of the two studies. In total, 386 first-time parents of 323 children were interviewed regarding their thoughts of their children’s screen habits when their first child was approximately 18 months old. The number of parents was greater than the number of children as, in some cases, both the mother and father of the same child were interviewed. Most participants were mothers (78%). The group of participants was heterogeneous regarding age (17–64 years) and length of education (2–23 years). The largest number of participants were born in Sweden (39%). Most interviews were conducted in Swedish (75%), followed by English. Approximately one in ten participants (11%) needed a language interpreter for the interview.

**Table 1:** Sociodemographic background factors of the participants

	*N*	%
**Child sex**		
Girl Boy	149 174	46.1 53.9
**Child age** in months (mean, range)	17.7 (14–23)	
**Parents’ role**		
Mother Father	300 86	77.7 22.3
**Parental age** (mean, range)	30.7 (17–64)	
**Education years** (mean, range)	14.6 (2–23)	
Region of birth*		
Sweden Europe (excl. Sweden) Sub-Saharan Africa MENA* Asia South and Central America	150 56 61 55 54 9	39.0 14.5 15.8 14.3 14.0 2.3
**Need of interpreter**		
Yes No	43 343	11.1 88.9
**Most common interview language**		
Swedish English Arabic Other	288 52 29 17	74.6 13.5 7.5 4.4

*One participant missing information

**MENA = Middle East and North Africa (Algeria, Bahrain, Egypt, the United Arab Emirates, Iran, Israel, Jordan, Kuwait, Lebanon, Libya, Morocco, Oman, Qatar, Saudi Arabia, Syria, Tunisia, Palestine, Yemen, and Turkey)

### Description of material

This study used selected data on screen use from the follow-up interviews of two larger studies. Two CHC centres were included in the first evaluation, and the follow-up data were collected from March 2019 to August 2020. At that time, two semi structured screen-related interview questions (Supplementary file 1), were developed and piloted by the first author (KTM), who is a professional paediatric nurse and a PhD candidate at the time of the interviews. The follow-up interviews from the other eight CHC centres were conducted from May 2021 to January 2022 by two paediatric nurses and four retired trained social workers and used the same questions (Supplementary file 1). All the interviewees had experience working with families in socioeconomically disadvantaged settings within the Stockholm region. The interviews were conducted using paper-based questionnaires and consisted of structured and semi structured questions.

The data were collected through phone interviews (*n* = 360), as this was the parents’ choice and because of the COVID-19 pandemic. One was conducted via email, and the remaining participants (*n* = 25) were interviewed at the CHC. If participants did not speak Swedish or English fluently, a language interpreter assisted in the interviews. An English version of the semi-structure questions addressed to the participating parents regarding children’s screen use can be found in Supplementary file 1. The interviewers were instructed to clarify the questions using their own words if needed.

Parents’ responses to the questions were not audio or video recorded. The interviewers wrote in the questionnaires a summary based on the participants’ responses, and afterwards, the responses were computed into a data file connected to codes given to each participant in the two studies. The length of the summarized responses to the addressed questions varied from 3 to 54 words (mean = 21).

### Data analyses

#### Quantitative analyses

SPSS 29 software was used for the descriptive analysis of the background characteristics of the participants’ frequencies, means and ranges. Quantitative analysis was conducted for the summarized, recorded interview responses, where the first author summarized and categorized parents’ responses related to thoughts about actual and appropriate age for introducing screens, children’s screen time per day, and type of screen. The results of those quantified categories are reported as frequencies and percentages.

#### Qualitative analysis

The qualitative analysis was conducted through content analysis inspired by Graneheim and Lundman’s manifest content analysis [23, 24]. The entire text material was read individually multiple times by the authors to build an overall understanding of the data. Thereafter the text material was scanned more closely to identify the meaning units in the material individually by each author. The meaning units were marked with different colours depending on the contents of the units. Thereafter, meaning units were condensed and coded. At the step of categorization, all authors discussed which categories would reflect the most central content that was said by the participants before establishing the final categories. These categories became the manifest content of the participants’ summarized responses.

## Results

### Young children’s screen use habits

The total number of participants in the study was *N* = 386. The results of the quantified analysis of children’s screen usage habits are displayed in Tables 2 and 3. The reported numbers and percentages in each table are based on the number of responses given to each variable that could be quantified in the data material.

**Table 2:** Children’s screen use at 15–18 months, including type of screen/s and duration

	*N*	%
**Screen use at the age of 15–18 months (N = 343)**		
Using screens at 15–18 months Not using screens at 15–18 months	317 26	92.4 7.6
**Type of screen (N = 317)**		
TV TV and tablet/phone Not specified which screen Tablet/phone	120 93 76 28	37.9 29.3 24.0 8.8
**Duration of screen time per day (N = 270)**		
Screen maximum 30 min Screen from 30–60 min Screen more than 60 min ^1^Little or short periods	131 19 47 73	48.5 7.0 17.4 27.0

^1^ “Little” or “short periods”: parents did not specify what/how many minutes this expression meant

**Table 3:** Parents responded about a suitable age for starting screen use and daily use of screens

	*N*	%
**When should children start screen use? (N = 206)**		
Before one year At two years (15–18 months too early) Later than at two years Postponed until no specific age	11 78 86 31	5.3 37.9 41.7 15.0
**Recommended duration of screen time per day (N = 82)**		
^1^Little or short periods Maximum 30 min More than 30 min	30 38 14	36.6 46.3 17.1

^1^ “Little” or “short periods”: parents did not specify what/how many minutes this expression meant

Approximately 92% of parents reported that their child had started to use screens by the age of 15–18 months. The most common kind of screen used was TV (Table 2). Furthermore, it was shown that most children used the screen for less than 30 min per day, and one group of parents (24%) did not specify the exact duration of screen use but described that their children used screens “little” or “for short periods” (Table 2).

Most parents who responded had an opinion that screens should be introduced when children are older than two years of age, followed by a group of parents with the opinion that screens could be introduced at the age of two years (or that it was too early to use screens at the age of 15–18 months). Another group of parents expressed that the introduction of screens should be postponed but without specifying the age. There were some parents among the participants who said that screens could be introduced before the age of one year (Table 3).

Most of the participants who responded to the question regarding suitable daily screen time expressed that it should not exceed 30 min, followed by a group who expressed that screen time should be limited to “little” or “short periods” without specifying what/how many minutes this expression meant. Approximately 17% reported durations exceeding 30 min when asked for suitable daily screen time for children (Table 3).

### First-time parents’ reflections related to children’s screen use

The qualitative analysis of first-time parents’ reflections on children’s screen use resulted in three main categories, each with two subcategories: ***screen use patterns****(screen-related time and reasons for screen use)*, ***perceived concerns with screen use****(child development and social issues)* and ***attitudes related to screen use****(parents’ attitudes towards screens and child’s response to screens)*. The results for each category are shown with short quotations of summarized notes written by the interviewers.

### Screen use patterns

Parents’ reflections on to what extent the children were allowed to use screens and why parents let them use screens.

#### Screen-related time

It appeared in parents’ reflections that screen use was not necessarily perceived as good for their young children. However, parents’ responses regarding screens showed that they allowed their young children to spend time with diverse types of screens (TV, smartphone, tablets) daily. TV was the first screen introduced by many and the dominating screen at 15–18 months (as described above) and was mentioned by parents to be a better alternative than other screens. Some parents described how they had introduced smartphones to their babies at the age of two to three months, while other parents had not yet introduced any screens at the time of the interview. Consequently, screen time, including TV and other screens, varied from no screen time to three hours per day. Some examples of this variation are given in these summarized responses from the parents:

*She is already using screens. It is difficult to say how much*,* maybe one hour per day.*

*He was three months when he started watching TV. He watches TV now*,* children’s programs*,* 10–20 min. He is interested*,* does not have a tablet*,* and does not touch the phone.*

*There is currently no TV or phone. At the age of three years*,* one hour per day will be fine.*

Overall, there was considerable variation in the participants’ responses regarding how much parents reported their children to be using diverse kinds of screens at the age of 15–18 months. Parents sometimes considered that the time spent on screens was too much for their young children. Even if the parents expressed that they had decided to set a time limit for their child’s screen use, they stated how difficult it was to maintain the limit in everyday life.

*The goal is to not use screens… using phone/tablet every second day*,* maximum 30 min.*

*Preferably not (screens) every day. It is better to wait as long as possible. In reality*,* she is watching TV*,* playing with the iPad sometimes…*.

Furthermore, the participating parents stated that children aged 15–18 months were too young for the screens and that they should be two years or older before they start using smartphones or tablets. In some cases, parents described that they let children’s interest control screen use, while others practiced a clear limit for screen use.

#### Reasons for screen use

The participating parents described how they used screens to do their own things without being disturbed by their child, to concentrate on a certain task or to have time for themselves. Some examples of parents’ responses are as follows:

*We let him watch when needed*,* for a maximum of 30 min*,* via either TV or phone.*

*We use tablets or phones when we meet with our friends.*

Screens showing films and YouTube were also mentioned to be used at mealtimes when child feeding problems existed or to calm mealtimes. Parents also described how screens could calm children, comfort them, and help them fall asleep at bedtime and brush their teeth. Additionally, parents described that screens were used to calm and distract children when travelling. As one parent expressed,

*We use the phone when we travel by car.*

Parents also mentioned that screens could contribute to children’s development and knowledge through pedagogical children’s programs and that using screens could strengthen children’s learning of their parents’ native language. One parent described it in the following way:

*She is learning a lot through different films and programs.*

Screens were also a way to stay connected and spend time with relatives living in other countries. Furthermore, a few parents expressed that it was important for children to learn how to use digital techniques at an early age.

There were parents who reflected that they had a habit of always having their TV on in the background. Parents also expressed that TV was used to spend time together with children to watch a children’s program. The importance of using screens together was mentioned and regarded as much easier when watching TV.

*We watch together for 15 min at a time. If we stay at home*,* we watch more.*

### Perceived concerns with screen use

Parents described that screen use in children of the relevant age group could entail many risks related to development and social issues.

#### Child development

Parents expressed several concerns about how screens could affect children’s motor, psychosocial, physical, and language development. Parents described young children’s obsession with screens and were worried that their children could develop an addiction to them. Furthermore, screens could have negative effects on children’s language development, ability to concentrate, imagination, and ingenuity. Regarding children’s physical health, parents mentioned concerns about children’s vision and physical inactivity. One parent expressed it the following way:

*Screens are not good for the eyes – nor for their concentration when they later start school.*

#### Social issues

Some of the parents felt that their children should not use screens and that screen use could have negative consequences but let their children use screens anyway since it was regarded as a societal norm. Furthermore, it was thought that it was important for children to learn to handle screens and digital devices at an early age to keep up with other children. Additionally, parents were observing what other parents did, and one parent expressed it in the following way:

*Other parents often give their children the phone and screens. It is not good.*

### Attitudes related to screen use

The parents’ responses revealed several attitudes regarding screen use. Furthermore, they described their children’s interest in using screens.

#### Parents’ attitudes towards screens

It was easy to have strong opinions about children’s screen use before having one’s own children. Moreover, parents’ attitudes towards screen use were not similar between friends and other parents who had children of the same age. There were also parents who mentioned that there were different opinions about children’s screen use in their own family, and screens could create fractions and trouble in a family if parents disagreed about screen use.

*It is a problem – we are fighting. The mother thinks for one hour – the father wants the screen to be on. This is a problem.*

Parents also expressed uncertainty and ignorance about screen use by using words that indicated that the subject was complicated or that screens were difficult.

*This is a tricky question. It is hard not to (use screens). I don’t know from what age. I don’t think it is good*,* wait until three or four years. What do they watch?*

Parents also reflected on their own use of screens and said that they avoided screen use when they were with their child.

*We try not to use our phones so often.*

Many parents wanted to postpone future screen use. However, there was greater acceptance of TV than of other screens. Parents further expressed that screens should not replace a child’s contact with adults, play time, or other activities such as drawing or having a walk outside.

#### Child’s response to screens

There were parents who described that their children did not show any interest in TV or other screens, or they focused for a brief time, while other parents expressed that their children were fully absorbed by the screens and protested when the screens were removed. It was further described that if the TV was on the background, the child could start dancing when hearing the music or interrupt their play to watch the TV. A few examples of parents’ reflections on their child’s responses to screens include the following:

*She losts interest (for screens) after 10 min.*

*He is obsessed with electronics. He steals his mum’s phone. He is interacting with the phone. There is no reason right now to impose limits*,* two hours*,* but shorter episodes when he becomes older.*

## Discussion

This study aimed to explore young children’s screen use habits and describes first-time parents’ reflections related to children’s screen use in socioeconomically disadvantaged Swedish settings.

The main findings of the study were that screen habits among the children varied, but most children used a screen daily, at least for shorter periods. Qualitative analysis of parents’ reflections on screen use resulted in three main categories: *screen use patterns*, *perceived concerns with screen use*, and *attitudes related to screen use* relating to the applicability of screens in everyday life, social norms, and concerns about screen use.

The main category of *screen use patterns* revealed that parents thought that there should be a time limit for screen use for their 15- to 18-month-old toddlers. This finding revealed that screen use may not be useful for young children. Parents could tell exactly what their reality looked like, at what age their child started to use screens, which kind of screens, and for how long screens were used daily. There were families who had zero screen time for their children, while for some, screen use could start as early as two to three months of age. Furthermore, there were toddlers who could spend up to three hours per day using screens.

These results are similar to those of previous studies exploring toddlers’ screen use, both because screen habits are well established at an early age [1–3] and because screen use can start when babies are just a few months old [4]. The variety of how long children use screens in this study may be explained by the reality that official recommendations of screen time are lacking in Sweden [9], and even the guidelines for nurses working within Swedish CHC services do not include a specific time limit for children’s screen use [12], while the WHO recommends zero screen time for children under one year [8]. However, even studies outside of Sweden have shown that guidelines regarding screen time recommendations by healthcare professionals are not always available to parents [13], and a lack of consensus on the recommendations parents receive is an obstacle to managing children’s screen time [7]. Additionally, both our study and a previous study demonstrated the struggle caused by the fact that screens are ingrained in daily routines and ways of life and cannot be removed, despite parents being aware of the possible negative health effects for their young children [15], and that this might still be the reality even if official recommendations were in place.

Both our study and previous studies revealed several *reasons for screen use*, including baby-sitting, educational means, and rewards [14]. In our study, parents also described how screens are used to handle situations in everyday life by distracting the child. Some of these recommendations, such as using screens while travelling, can be found in the recommended advice that CHC nurses can give to parents [12]. At the same time, parents in this study described the use of screens at mealtimes and before going to sleep, which are examples of situations where screens should be avoided according to the recommendations of Swedish CHC nurses [12]. Furthermore, some parents also reported using screens to calm down their children. Findings from a recent study suggest that using screens to distract or alleviate distress among young children may work detrimentally to their own abilities to regulate emotions, although more research is needed to confirm those findings [25]. Screens were also used by parents in this study to gain time for themselves and to be able to focus on certain things, described as ‘baby-sitters’ in previous research [14]. It seems that families use screens in a way that makes their everyday life easier, where short-term solutions are prioritized over potential long-term consequences. At the same time, using distraction to get a break or help in parenting could be seen as beneficial for the parental role. Furthermore, this study could identify possible benefits of children’s screen use such as viewing TV together with a child or learning from the pedagogical children’s programs and apps, also seen in previous studies [26]. Even connecting with relatives with extended family overseas was described in this study which is known to be beneficial for infants and toddlers [27].

Regarding our second main category, *perceived concerns with screen use*, parents described social issues in part, showing that even if they thought that screens might not be good for their children, children used screens anyway, as parents saw it as a societal norm. This may be related to the fact that screens are connected so deeply to our daily lives that they cannot be easily removed from adult routines, despite knowledge of possible negative consequences [15]. Furthermore, parents described that children’s screen use could create fractions in relations if both parents did not agree about the child’s screen use patterns. This is important knowledge for professionals who meet families with young children and discuss screen use, for example, CHC nurses, and could underline the importance of trying to discuss screen habits when both parents are present.

The fact that the parents of this study were aware of the negative effects that screens could have on their child’s development is in accordance with previous studies, including physical, behavioural, and psychosocial aspects [6]; language; and cognitive development and communication [7]. The parents in this study were also concerned about their children’s vision and physical inactivity. Further concerns such as negative effects on children’s ability to concentrate and on imagination and ingenuity were also mentioned. These findings confirm that parents are aware of the consequences related to children’s screen use, as described in previous research [6, 7].

Our third main category, *attitudes related to screen use*, reveals that participating parents have different attitudes about children’s screen use, sometimes even within the same family. It was expressed that it was easy to have certain attitudes about screen use until one became a parent. Some parents reflected on their own screen use and on not using it when they were with their child. This is an interesting finding, as it is known that children’s screen use and parents’ own screen time are associated [16]. Furthermore, in the Swedish context, CHC nurses are advised to help parents reflect on their own use of screens [12]. This finding may be worth further exploration regarding whether parents’ self-reflections about their own screen use, together with some guidelines including recommended time limits for screens, might be a way forward regarding toddlers’ healthy screen use.

The participating parents described how their children seemed to be addicted to screens and wanted to use screens more. However, there were parents who described their children as not interested in screens. These findings are interesting; there might be many reasons why some children are drawn to the screens while others are not interested, and this may awaken interest for future research about why this is so. Previous research has described several aspects associated with children’s screen use, including socioeconomic background factors [17]. Our study was conducted in socioeconomically disadvantaged settings, and differences in children’s responses were found, which may indicate that children’s personality and character can impact their interest in screens, as well as families’ ways of activating their children.

### Strengths and limitations

One of the strengths of this study is that it included many participants from socioeconomically disadvantaged settings. Furthermore, the sample is heterogeneous in the sense that it includes both mothers and fathers and Swedish- and foreign-born parents of different ages and education levels which increases the chances of the findings being transferable to similar settings in urban areas. Likewise, our study managed to collect quantifiable data about children’s screen use, which provides background information for parents’ further reflections.

However, a few limitations related to our study are identified. One relates to the fact that parents’ responses to the questions regarding screen use were not audio recorded, were merely summarized, and were noted by interviewers. This may have led to a loss of information, as interviewers may have focused on certain responses given by parents to summarize the responses in a written way. However, the first author (KTM), who piloted the questions and wrote notes based on parents’ responses, provided a thorough introduction to the other interviewers regarding how the interview questions would be presented and the responses noted, as audio recordings were not used. Nevertheless, written notes about participants’ responses tended to describe from which age and for how long screens were used by participants’ children, accompanied by reflections, rather than responding to at what age and for how long screen use would be suitable. Parents’ responses contributed to an informative picture of the parents’ reality of screens in their everyday lives. Furthermore, the summarized responses overall tended to focus on the negative descriptions of children’s screen use, which can be related to how questions were asked, and the summarized responses interpreted and noted by interviewers. However, the interviewers were instructed beforehand to strive to keep a positive attitude during the interviews to explore children’s screen use through parents’ responses. Indeed, positive features with screen use such as were also described by the parents which indicates an allowing ambiance during the interviews, and it might be that the negative descriptions are reflecting the parents’ negative attitudes towards their children’s screen use. Furthermore, parents’ reflections can be regarded as an identified expression of their need to discuss children’s screen use with professionals. However, not all participants responded to all the questions, which are reported as variables related to children’s screen habits in this study. The percentage of parents who reported children’s screen use at 15–18 months, including type of screen/s and duration, was high (79–89%), while the number of participants who responded about the appropriate age for starting screen use and daily use of screens remained low (21–53%).

Another limitation is that a large share of the interviews was conducted using interpreters or in a language other than the first language of the parents, which increases the risk of misunderstandings. The interviewers were all familiar with meeting foreign-born parents from their prior work experience and did their best to accommodate these parents and double check whenever their responses were unclear. One last limitation is related to the transferability of the study, as all CHCs included were in the region of Stockholm. The findings might differ in disadvantaged settings located more rurally or even in other large city areas. However, the sample’s heterogeneity increases the chances of capturing diverse perspectives that could also be applicable to other similar settings.

## Conclusions

First-time parents in socioeconomically disadvantaged settings expressed awareness of possible negative screen-related effects and recommendations but did not always focus on long-term consequences when using screens in everyday life. Screens used as a short distraction, time spent together with screens, infants’ contact with relatives overseas, and pedagogical children’s programs and apps can be regarded as screen-related benefits. Encouraging parents’ self-reflection on their own screen use may be a way of contributing to healthier screen habits among young children. Future studies are suggested regarding parents’ and children’s excessive interest in screens and how to manage screen use as a family.

### Electronic supplementary material

Below is the link to the electronic supplementary material.

Supplementary Material 1

## Data Availability

The data generated and/or analysed in the current study are not publicly available due to ethical approval (registration no. 2017/1587-31/5; registration no. 2017/1587-31/5; 2019–04086 and 2021–01984), which states that the use of interview data should respect the anonymity of participants, only be accessed by the research group, and be stored locked by passwords. The corresponding author can be contacted for further information.

## Abbreviations

CHC: Child Health Care
CNI: Care Need Index
KI: Karolinska Institutet
WHO: World Health Organization
