# Supplementary material for: Young children’s screen habits and first-time parents’ reflections on screen use in socioeconomically disadvantaged Swedish settings: a mixed methods study
Source: BMC Public Health. 2024 Jul 29;24:2027. doi: 10.1186/s12889-024-19557-9 (PMC11285388; doi:10.1186/s12889-024-19557-9)
Supplement: Supplementary file 1 — Supplementary Material 1 [file 12889_2024_19557_MOESM1_ESM.docx]

Supplementary file 1.

The following two questions addressed to the participating parents regarding children’s screen use:

1. *At what age do you think it is appropriate for your child to start watching TV, using a tablet or a mobile phone?*
2. *For how long should a child watch TV or use a tablet or a mobile phone?*
